# Correction: Gill, R.D. Does Geometric Algebra Provide a Loophole to Bell’s Theorem? *Entropy* 2020, *22*, 61

**DOI:** 10.3390/e23050631

**Published:** 2021-05-19

**Authors:** Richard David Gill

**Affiliations:** Mathematical Institute, Leiden University, Rapenburg 70, 2311 EZ Leiden, The Netherlands; gill@math.leidenuniv.nl

Corrections are made to my paper “Gill, R.D. Does Geometric Algebra Provide a Loophole to Bell’s Theorem? *Entropy* 2020, *22*, 61”. Firstly, there was an obvious and easily corrected mathematical error at the end of Section 6 of the paper. In the Clifford algebra under consideration, the basis bivectors $Me_{i}$ do not square to the identity, but to minus the identity. However, the trivector *M* does square to the identity and hence non-zero divisors of zero, M−1 and M+1, can be found by the same argument as was given in the paper.

Secondly, in response to a complaint about *ad hominem* and *ad verecundam* arguments, a number of scientifically superfluous but insulting sentences have been deleted, and other disrespectful remarks have been rendered neutral by omission of derogatory adjectives. I would like to apologize to Dr. Joy Christian for unwarranted offence.

The end of Section 6 of Gill (2020) [1] discussed the even sub-algebra of $C\ell_{4,0}$, isomorphic to $C\ell_{0,3}$:

One can take as basis for the eight-dimensional real vector space $C\ell_{0,3}$ the scalar 1, three anti-commuting vectors $e_{i}$, three bivectors $v_{i}$, and the pseudo-scalar $M=e_{1}e_{2}e_{3}$. The algebra multiplication is associative and unitary (there exists a multiplicative unit, 1). The pseudo-scalar *M* squares to −1. Scalar and pseudo-scalar commute with everything. The three basis vectors $e_{i}$, by definition, square to −1. The three basis bivectors $v_{i}=Me_{i}$ square to +1. Take any unit bivector *v*. It satisfies $v^{2}=1$ hence $v^{2}-1=\left(v-1\right)\left(v+1\right)=0$. If the space could be given a norm such that the norm of a product is the product of the norms, we would have ∥v−1∥.∥v+1∥=0 hence either ∥v−1∥=0 or ∥v+1∥=0 (or both), implying that either v−1=0 or v+1=0 (or both), implying that v=1 or v=−1, neither of which are true.

But the bivectors $v_{i}$ square to −1 and the trivector *M* squares to +1. Still, it then follows that (M+1)(M−1)=0, and by the argument originally given, it follows that M=1 or M=−1, a contradiction.
